# 4H-SiC Double Trench MOSFET with Split Heterojunction Gate for Improving Switching Characteristics

**DOI:** 10.3390/ma14133554

**Published:** 2021-06-25

**Authors:** Jaeyeop Na, Jinhee Cheon, Kwangsoo Kim

**Affiliations:** Department of Electronic Engineering, Sogang University, Seoul 04107, Korea; thekal75@naver.com (J.N.); tofawk@naver.com (J.C.)

**Keywords:** 4H-SiC, double trench MOSFET, split gate, gate charge, body diode, heterojunction, reverse recovery charge, switching loss

## Abstract

In this paper, a novel 4H-SiC split heterojunction gate double trench metal-oxide-semiconductor field-effect transistor (SHG-DTMOS) is proposed to improve switching speed and loss. The device modifies the split gate double trench MOSFET (SG-DTMOS) by changing the N^+^ polysilicon split gate to the P^+^ polysilicon split gate. It has two separate P^+^ shielding regions under the gate to use the P^+^ split polysilicon gate as a heterojunction body diode and prevent reverse leakage `current. The static and most dynamic characteristics of the SHG-DTMOS are almost like those of the SG-DTMOS. However, the reverse recovery charge is improved by 65.83% and 73.45%, and the switching loss is improved by 54.84% and 44.98%, respectively, compared with the conventional double trench MOSFET (Con-DTMOS) and SG-DTMOS owing to the heterojunction.

## 1. Introduction

Silicon carbide (SiC) is a wide bandgap material, and 4H-SiC metal-oxide-semiconductor field-effect transistor (MOSFET) is one of the most promising semiconductors in high-power systems owing to its high breakdown voltage (BV), high critical electric field, and high thermal conductivity [1,2].

Trench MOSFETs are one of the preferred device structures because of their low specific on-resistance (R_on-sp_); however, a high electric field on the gate oxide and a large gate-drain capacitance (C_rss_) degrade the breakdown voltage characteristics and the switching performance. To alleviate this problem, a double trench MOSFET (DTMOS) with a P^+^ shielding region structure has been developed [3,4,5]. The double trench structure with a P^+^ shielding region distributes the high electric field of the gate oxide to P^+^ type doping on the source and gate regions. Therefore, it improves the breakdown voltage characteristics of the device [6]. Its gate P^+^ shielding region also reduces the charge coupling effect between the gate and drain so that the C_rss_ and gate-drain charge (Q_GD_) are significantly reduced and have better switching performance [7]. Currently, a split-gate double trench MOSFET (SG-DTMOS) with a better high-frequency FOM (R_on-sp_ × Q_GD_) compared with the conventional DT MOSFET (Con-DTMOS) has been proposed [8,9]. An SG-DTMOS is a structure in which the upper and lower gates are separated, the upper gate is in contact with the gate voltage, and the lower gate is in contact with the source voltage. When the area of the active channel gate is reduced, it has a slightly higher R_on-sp_ and a significantly reduced C_rss_ and Q_GD_ compared with the Con-DTMOS; hence, its switching performance is better than that of the Con-DTMOS.

Power electronic module systems, such as inverters and converters, require freewheeling diodes and SiC Schottky barrier diodes (SBDs), which are widely used as anti-paralleled SiC MOSFETs [10]. A SiC SBD has a relatively low forward voltage drop and almost no reverse recovery charge. Embedded PiN diodes in SiC MOSFETs can also be used as freewheeling diodes, but they have a large forward voltage drop and a large reverse recovery charge and time; therefore, power module systems with SiC SBDs result in better switching losses [11].

However, an external SBD leads to a higher die cost and a larger assembly area in the power module system [12]. To solve this problem, a SiC MOSFET with an embedded SBD has been proposed, which integrates SiC SBD and SiC MOSFET in one chip [13]. Nevertheless, a large reverse leakage current can occur due to the image charge of the metal-semiconductor junction and contamination by the metal for the Schottky contact [14]. Recently, studies on embedded heterojunction body diode MOSFETs have been conducted, but high leakage current and low critical electric field of polysilicon make it difficult to operate at high voltage [15,16].

In this paper, a novel 4H-SiC split heterojunction gate double trench MOSFET (SHG-DTMOS) is proposed, which replaces the N^+^ polysilicon source-contacted split gate region of SG-DTMOS with a P^+^ polysilicon split gate. By this, the split gate part can be used as a body diode while reducing the gate charge still very effectively. It also has a separate gate P^+^ shielding region to conduct the heterojunction body diode in the forward bias condition and effectively block the reverse leakage current and high electric field in the off-state condition. Therefore, SHG-DTMOS is suitable for high-voltage operations. Embedded heterojunction diodes in MOSFETs behave like SBDs in freewheeling diodes. Consequently, the SHG-DTMOS has a superior switching speed and switching loss with a small gate charge and low reverse recovery charge, exhibiting excellent body diode characteristics.

The research was simulated using TCAD Sentaurus simulation with ver. 0-2018.06. 2-D mixed-mode simulation considered for circuit simulation. The electron/hole continuity and Poisson equations were solved, and the Okuto-Crowell avalanche, incomplete ionization, Shockley–Read–Hall recombination, and Auger recombination were used for avalanche and recombination models [17]. In addition, the Lombardi mobility model, doping dependency, high field saturation, appropriate fixed charge, and interface trap charge were considered to match the channel mobility about 30~40 cm^2^/(V∙s) [18,19]. Bandgap narrowing, anisotropic material properties were also considered for simulation. All simulations were set to 1 cm^2^ of the active area, and the temperature was set to 300 K.

## 2. Proposed Device Structures

**Figure 1:**
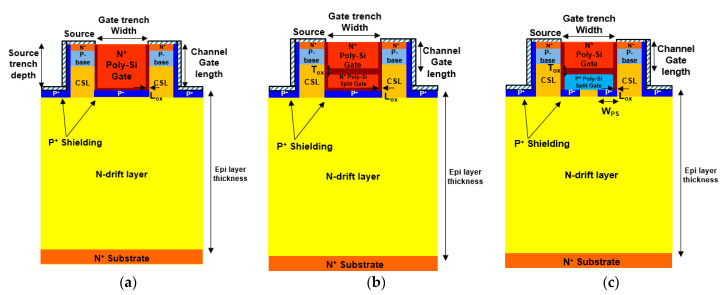
Schematic cross-sectional view of (**a**) Conventional Double Trench MOSFET (Con-DTMOS), (**b**) Split Gate Double Trench MOSFET (SG-DTMOS), and (**c**) Split Heterojunction Gate Double Trench MOSFET (SHG-DTMOS).

Figure 1 shows a cross-sectional schematic diagram of (a) Con-DTMOS [5], (b) SG-DTMOS, and (c) SHG-DTMOS. All the devices have a double trench structure, with a current spread layer (CSL) region that helps the current spread well [20]. They also have a source trench P^+^ shielding region and a gate trench P^+^ shielding region, which are shorted with the source electrode. Compared with the Con-DTMOS, the SG-DTMOS and SHG-DTMOS both have source-contacted split gate regions, which greatly reduce the C_rss_ by decreasing the gate-drain coupling effect [8]. The SHG-DTMOS has a P^+^ polysilicon split gate, whereas the SG-DTMOS only has an N^+^ polysilicon gate. This P^+^ polysilicon is in direct contact with the CSL region and the separate gate P^+^ shielding regions. When a forward bias is applied, the current flows from the P^+^ polysilicon to the CSL and N-drift region owing to its low turn-on voltage. Therefore, this embedded heterojunction diode acts like an SBD.

The cell pitch of all structures is 6 μm, and the width of the gate trench is 2 μm. The epi-layer thickness of all the devices is 13 μm, and the doping concentration of all the structures is the same, except for the P^+^ polysilicon of the SHG-DTMOS. The doping concentration of the P^+^ polysilicon in the SHG-DTMOS is 5 × 10^18^/cm^3^. Additional parameters of the structures are listed in Table 1.

## 3. Results and Discussion

### 3.1. Static and Dynamic Characteristics

**Figure 2:**
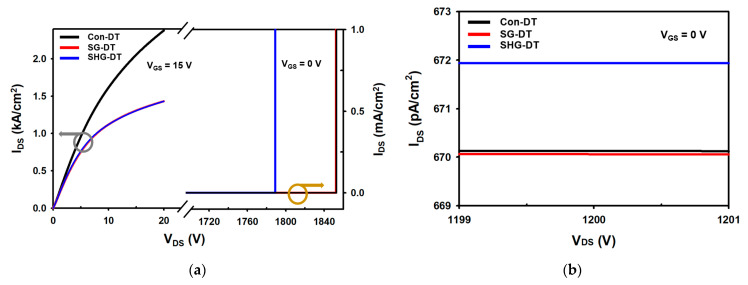
Comparison of Con-DTMOS, SG-DTMOS, and SHG-DTMOS. (**a**) Specific on-resistance and breakdown voltage. (**b**) Reverse leakage current.

For static characteristics, the breakdown voltage (BV), specific on-resistance (R_on-sp_), maximum oxide electric field (E_mox_), and reverse leakage current of the devices were simulated. Figure 2a shows the I–V characteristics of the devices. The R_on-sp_ values are 4.74 mΩ∙cm^2^, 5.55 mΩ∙cm^2^, and 5.45 mΩ∙cm^2^, whereas the breakdown voltages are 1853 V, 1852 V, and 1789 V for the Con-DTMOS, SG-DTMOS, and SHG-DTMOS at I_DS_ = 100 μA/cm^2^, respectively [21]. The SG-DTMOS and SHG-DTMOS have slightly higher R_on-sp_ values than the Con-DTMOS because of the split gate. The reduced active channel gate decreases the accumulation layer, and the depletion region by the split gate disturbs the current path, such that the specific on-resistances of the SG-DTMOS and SHG-DTMOS are slightly larger [8,22]. The breakdown voltage of the SHG-DTMOS slightly decreases because of the separate gate P^+^ shielding regions and the P^+^ polysilicon, but the difference is not large compared with the other two devices.

**Figure 3:**
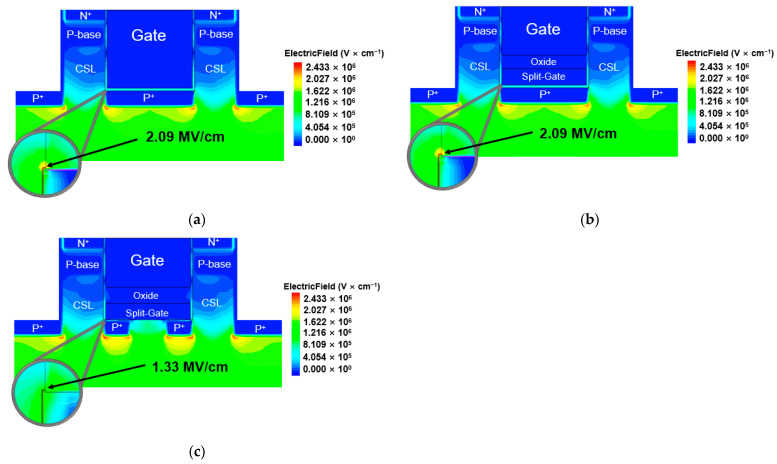
Schematic cross-sectional view of electric field distribution at V_DS_ = 1200 V (**a**) Con-DTMOS, (**b**) SG-DTMOS, and (**c**) SHG-DTMOS.

Figure 2b shows the reverse leakage current of the off state at V_DS_ = 1200 V, and Figure 3 shows the off-state electric field distribution of each device at V_DS_ = 1200 V. The electric field intensity applied to the P^+^ polysilicon and the magnitude of the reverse leakage current are very important in heterojunction devices because the critical electric field and the bandgap of the polysilicon are smaller than those of SiC [23]. In Figure 3, the maximum electric field applied to the P^+^ polysilicon split gate of the SHG-DTMOS is 0.43 MV/cm, and the maximum electric field between the P^+^ polysilicon and the CSL junction is 0.34 MV/cm, which is lower than the critical electric field of highly doped silicon [24,25]. In addition, in Figure 2b, the reverse leakage current of the SHG-DTMOS is slightly larger than those of the other two devices, but there is minimal difference. This is due to the separate gate P^+^ shielding region. When the source biased zero and a large bias is applied to the drain, the two separate gate P^+^ shielding regions fully deplete the CSL region under the split gate. This depletion region blocks the P^+^ polysilicon from a high drain voltage, reducing the maximum electric field of the P^+^ polysilicon and the reverse leakage current, such as the junction barrier Schottky (JBS) [26]. Figure 3 shows that the maximum electric field of the gate oxide (E_mox_) of all devices does not exceed 3 MV/cm. Therefore, the SHG-DTMOS, such as the other two devices, has a low reverse leakage current and a high gate oxide reliability even at a high operating voltage of 1200 V or higher.

**Figure 4:**
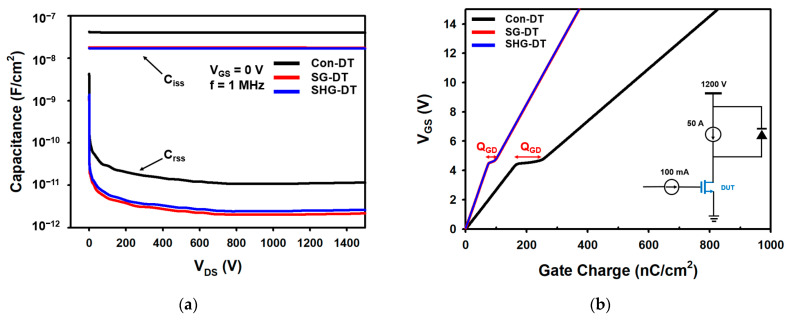
Comparison of Con-DTMOS, SG-DTMOS, and SHG-DTMOS of (**a**) C_iss_ and C_rss_. (**b**) Gate charge.

Figure 4a shows the gate-drain capacitance (C_rss_) and the input capacitance (C_iss_: C_iss_ = C_gs_ + C_rss_) of the devices. The gate voltage was fixed at 0 V, the small AC signal was 1 MHz, and the drain voltage was swept from 0 to 1500 V. Because the SG-DTMOS and the SHG-DTMOS have short active channel gates, the gate-drain overlap region is reduced, which is the reason for the ultralow C_rss_ compared with the Con-DTMOS. The reduction in the channel gate length also affects the overlap region between the gate and source. Therefore, it can be observed that the C_iss_ of the two devices with split gates are also smaller than that of the Con-DTMOS.

Figure 4b shows the gate charges of the Con-DTMOS, SG-DTMOS, and SHG-DTMOS. Because the gate charge is proportional to the gate capacitance, the SG-DTMOS and SHG-DTMOS have ultralow gate charge (Q_G_) and gate-drain charge (Q_GD_) compared with the Con-DTMOS. Compared with the Con-DTMOS, the Q_GD_ of the SG-DTMOS and SHG-DTMOS decreased by 73% and 71%, respectively. The SG-DTMOS and SHG-DTMOS have a smaller high-frequency figure of merit (HF-FOM) owing to the reduced Q_GD_, even though the R_on-sp_ is slightly larger than that of the Con-DTMOS. From these results, it is evident that there is no significant difference in the performance of the SHG-DTMOS compared with the SG-DTMOS despite slight structural changes. The overall characteristics are summarized in Table 2.

### 3.2. Body Diode and Switching Characteristics

**Figure 5:**
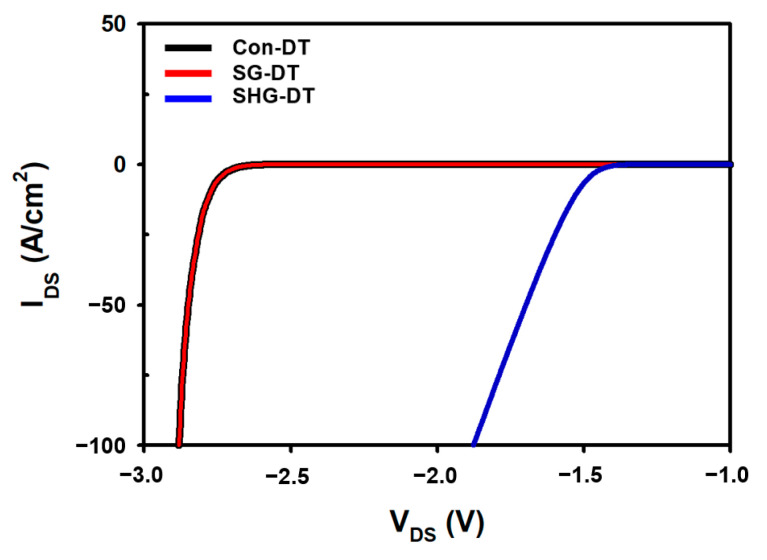
The forward conduction state characteristics of body diodes of Con-DTMOS, SG-DTMOS, and SHG-DMOS.

**Figure 6:**
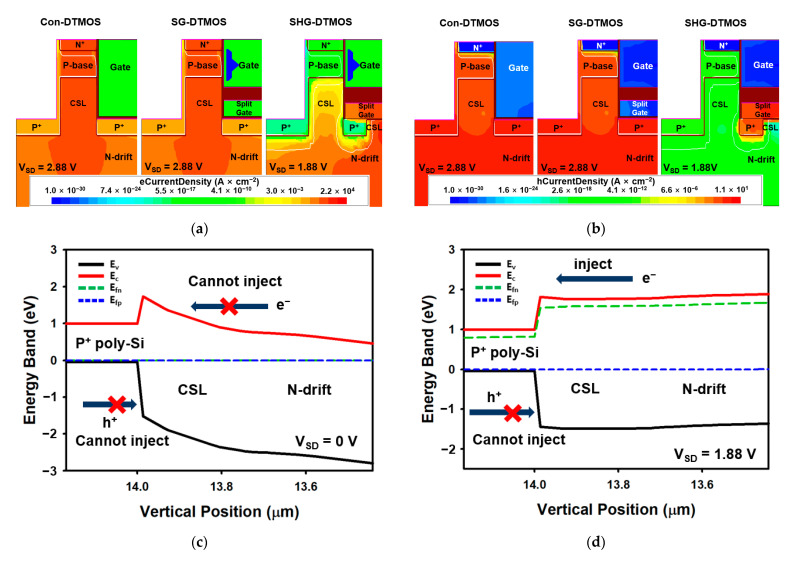

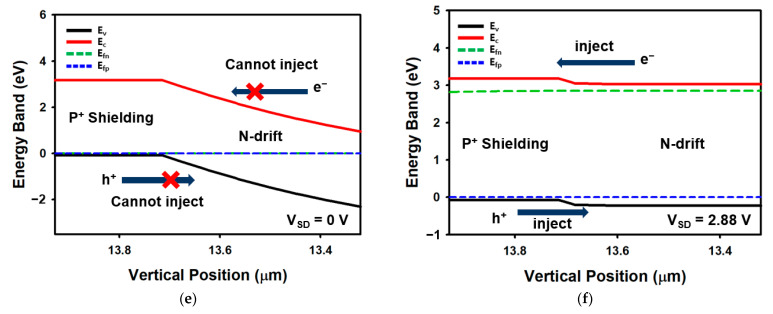
(**a**) The electron current distribution of Con-DTMOS, SG-DTMOS, and SHG-DTMOS. (**b**) The hole current distribution of Con-DTMOS, SG-DTMOS, and SHG-DTMOS. (**c**) The band diagram of SHG-DTMOS at V_SD_ = 0 V. (**d**) The band diagram of SHG-DTMOS at V_SD_ = 1.88 V. (**e**) The band diagram of SG-DTMOS at V_SD_ = 0 V. (**f**) The band diagram of SG-DTMOS at V_SD_ = 2.88 V.

The Con-DTMOS, SG-DTMOS, and SHG-DTMOS all have built-in PiN diodes in their device structure, which can be used as body diodes [27]. Figure 5 shows a comparison of the body diode characteristics of the Con-DTMOS, SG-DTMOS, and SHG-DTMOS in the forward conduction states. The forward conduction voltage V_F_ of the SHG-DTMOS at a current density of 100 A/cm^2^ is 1.88 V, which is approximately 34% smaller than the 2.88 V of the Con-DTMOS and the SG-DTMOS, respectively. This low forward conduction voltage results in a lower dead-time energy loss [28]. Figure 6a,b show the electron and hole current distributions of each device at the forward voltage. Unlike the Con-DTMOS and SG-DTMOS, it can be observed that there is almost no hole current flowing from the source to drain in the SHG-DTMOS. This is related to the barrier height between the P^+^ polysilicon and SiC. Figure 6c,d shows the band diagram of the SHG-DTMOS. The band diagram in Figure 6 shows the energy band according to the device height at the horizontal center region of the device. When there is no bias, the energy barrier is high, so both electrons and holes cannot move, and no current flows. When a forward bias is applied, the height of the energy barrier is lowered, and the lowered barrier allows electrons to move from the N-drift region to the P^+^ polysilicon. On the other hand, the energy barrier height in the hole is still high; thus, the hole cannot move from the P^+^ polysilicon to the N-drift region. Therefore, minority carrier injection in the drift region does not occur, and no hole current flows. In Figure 6e,f, however, when a forward bias is applied to the SG-DTMOS, the energy barrier height is lowered so that hole and electrons can flow sufficiently, and minority carrier injection occurs in the drift region.

**Figure 7:**
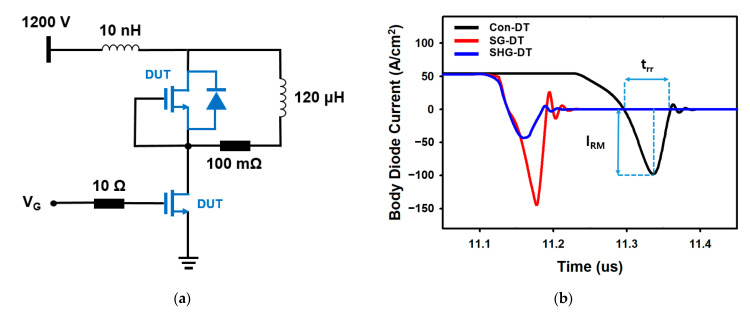
(**a**) Test circuit of reverse recovery charge and switching characteristics. (**b**) Comparison of body diodes reverse recovery characteristics of Con-DTMOS, SG-DTMOS, and SHG-DTMOS.

Figure 7a shows the double pulse test (DPT) circuit with a body diode to evaluate the reverse recovery charge and time, and Figure 7b shows the reverse recovery current of the body diodes of the Con-DTMOS, SG-DTMOS, and SHG-DTMOS. When the body diode is switched from the on-state to the off state, in order to completely turn off, all the carriers injected inside must be swept away. During this process, a large reverse recovery current occurs, as shown in Figure 7b [29]. The reverse recovery charge (Q_rr_) is proportional to the reverse recovery time (t_rr_) and the peak current (I_RM_) [30]. In Figure 7b, the Qrr of the SHG-DTMOS is 1010 nC/cm^2^, which is 65.83% and 73.45% lower than those of the Con-DTMOS and SG-DTMOS, respectively. The absence of minority hole carrier injection significantly reduces the t_rr_ and I_RM_ of the SHG-DTMOS, resulting in a smaller Q_rr_ than the Con-DTMOS and SG-DTMOS.

**Figure 8:**
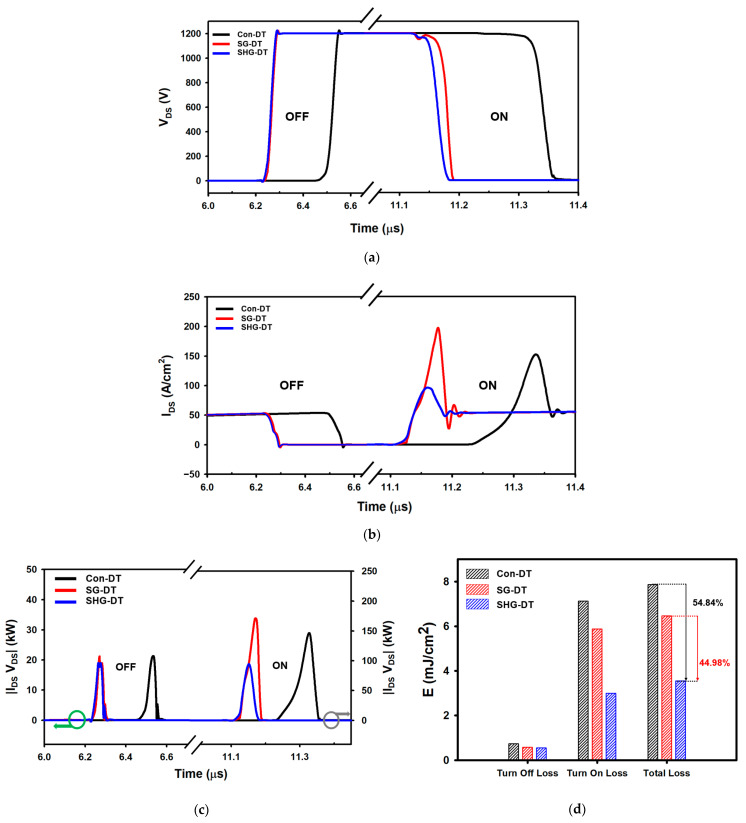
Comparison of Con-DTMOS, SG-DTMOS, and SHG-DTMOS of (**a**), (**b**) switching characteristics, (**c**) power consumption, and (**d**) switching loss.

Figure 8a,b shows a comparison of the turn-off and turn-on switching characteristics. The switching behavior of the MOSFET was simulated using the DPT circuit in Figure 7a. Switching on time T_on_ (T_on_: T_on_ = T_d,on_ + T_r_) and off time T_off_ (T_off_: T_off_ = T_d,off_ + T_f_) are defined as follows [31], where T_d,on_ is the turn-on delay time (from 10% of V_GS_ to 90% of V_DS_), and Tr is the rise time (from 90% to 10% of VDS at the falling edge). T_d,off_ is the turn-off delay time (from 90% of V_GS_ to 10% of V_DS_), and Tf is the falling time (from 10% to 90% of V_DS_ at the rising edge). The smaller gate charge reduced the total switching time of the SG-DTMOS and SHG-DTMOS by 50.13% and 51.9%, respectively, compared with that of the Con-DTMOS. The SHG-DTMOS’s turn-on overshoot of the drain current is very small compared with the Con-DTMOS and SG-DTMOS owing to the heterojunction body diode. Figure 8c,d show a comparison of the power loss and total switching loss of the Con-DTMOS, SG-DTMOS, and SHG-DTMOS. The SG-DTMOS has a short switching time compared with the Con-DTMOS because of the low gate charge, but the difference in the switching energy loss from the Con-DTMOS is not that much owing to poor body diode characteristics. The SHG-DTMOS shows a similar switching time as the SG-DTMOS, but it exhibits excellent body diode characteristics using the heterojunction diode, and the switching energy loss is significantly reduced by 54.84% and 44.98% compared with those of the Con-DTMOS and SG-DTMOS, respectively. Table 3 summarizes the body diode and switching characteristics of the three devices.

### 3.3. Optimization and Proposed Fabrication Process

**Figure 9:**
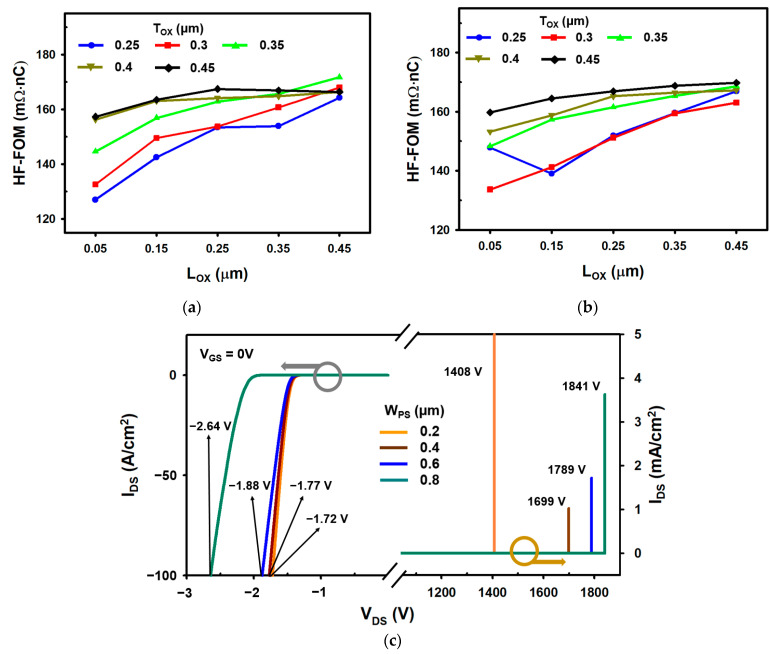
(**a**) HF-FOM of SG-DTMOS with varied L_ox_ and T_ox_. (**b**) HF-FOM of SHG-DTMOS with varied L_ox_ and T_ox_. (**c**) Influence of W_PS_ on forward conduction characteristics and breakdown voltage of SHG-DTMOS.

The length of the split gate side oxide L_ox_ and the thickness of the oxide T_ox_ between the two gates affect the characteristics of the SG-DTMOS and SHG-DTMOS [8,9]. As the length L_ox_ decreases, the effect of depletion by the split gate in the CSL increases, and hence, the specific on-resistance of the device increases. However, as the overlap area between the gate-drain decreases, the gate charge decreases. For the same reason, as T_ox_ decreases, R_on-sp_ increases, and the gate charge decreases. Therefore, it is important to properly adjust L_ox_ and T_ox_ to determine the optimal device. Figure 9a,b shows the HF-FOM of the SG-DTMOS and SHG-DTMOS, respectively, according to L_ox_ and T_ox_. In Figure 9a,b, the HF-FOM tendencies of SG-DTMOS and SHG-DTMOS are slightly different. Since the doping type of the split gate is changed from N^+^ to P^+^, the depletion region of SHG-DTMOS expands slightly wider than that of SG-DTMOS in the CSL region. This depletion region narrows the current path, which increases the R_sp-on_. For this reason, in Figure 9b, the HF-FOM at L_ox_ is 0.05 μm, and T_ox_ is 0.25 µm large, which deviates from the tendency. After that, if L_ox_ or T_ox_ increases, the current disturbance effect due to the depletion region is reduced, and the trend is again followed. In the SG-DTMOS, HF-FOM is smallest when L_ox_ = 0.05 μm and T_ox_ = 0.25 μm, whereas L_ox_ = 0.05 μm and T_ox_ = 0.3 μm in the SHG-DTMOS.

Figure 9c shows the effect of BV and V_F_ of SHG-DTMOS according to the separate gate P^+^ shielding width (W_PS_). As W_PS_ decreases, the area where the drift region (CSL) and the P^+^ split polysilicon are in contact becomes wider; therefore, more current can flow, and V_F_ decreases. The slope of the curve also steeps because the smaller W_PS_ has a lower diode on-resistance value. However, as the gate P^+^ shielding region decreases, the area of the P^+^ polysilicon exposed to the drain becomes wider, and BV decreases. When W_PS_ increases, the opposite result is obtained. In Figure 9c, choosing when W_PS_ = 0.6 μm is an appropriate approach in terms of V_F_ and BV. At W_PS_ of 0.6 μm, the increase in V_F_ is not large, indicating an acceptable BV value.

**Figure 10:**
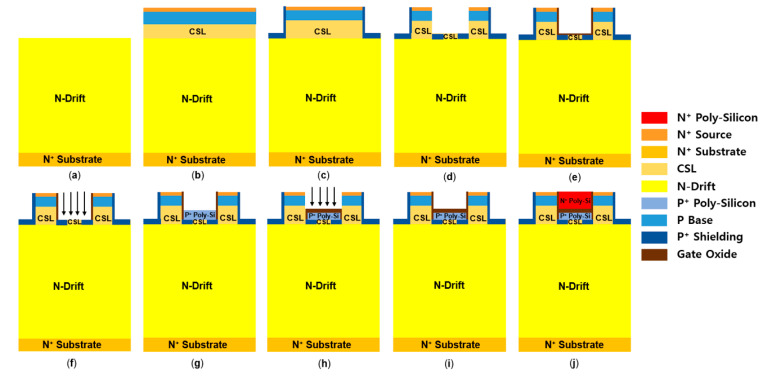
Proposed fabrication process of SHG-DTMOS. (**a**) Epitaxial N-drift layer on N^+^ substrate. (**b**) Form the CSL by epitaxial growth and form the P base region and N^+^ source region by double implantation. (**c**) Trench source region and P^+^ tilt ion implantation. (**d**) Trench gate region and form the separate P^+^ gate shielding region by ion implantation. (**e**) Thermal oxidation to form gate oxide. (**f**) Etch the bottom gate oxide by RIE-ICP etching. (**g**) Deposit P^+^ polysilicon and etch using the etch-back process and RIE-ICP. (**h**) Deposit oxide by CVD and etch the oxide. (**i**) Form sidewall gate oxide by dry thermal oxidation. (**j**) Deposit N^+^ polysilicon and etch back.

The fabrication process of the SHG-DTMOS is almost the same as that of the SG-DTMOS, except for the formation of separate gate P^+^ shielding regions and etching of the bottom split gate oxide. Figure 10 shows the fabrication process of the SHG-DTMOS. First, the N-drift region is formed by epitaxial growth on the N^+^ substrate, as shown in Figure 10a. Then, the CSL region is formed by epitaxial growth. The P base region is then formed with aluminum and the N^+^ source region with nitrogen by double implantation, as shown in Figure 10b [32]. Subsequently, the source region is trenched, and the source P^+^ shielding region is formed by tilt ion implantation. The gate region is trenched, and the separate gate P^+^ shielding region is implanted, as shown in Figure 10c,d [33]. Dry thermal oxidation is performed, and the bottom oxide is etched, as shown in Figure 10e,f. In Figure 10g, a P^+^ polysilicon split gate is deposited on the gate trench, and it is created to the desired length using the RIE-ICP etching process after the etch-back process [34]. Then, oxide is deposited by CVD, and etching is performed to produce a thick bottom oxide, as shown in Figure 10h [35]. Another dry thermal oxidation process is performed to make the sidewall gate oxide as shown in Figure 10f; finally, N^+^ polysilicon is deposited, and a channel gate is formed using the etch-back process, as shown in Figure 10j. Because the P^+^ polysilicon split gate of the SHG-DTMOS is in contact with the P^+^ gate shielding region, there is no need to make additional source contact with the split gate [36].

## 4. Conclusions

In this paper, a novel 4H-SiC split heterojunction gate double trench MOSFET (SHG-DTMOS) with characteristics that are like those of the SG-DTMOS, but with much-improved switching loss in the body diode, is proposed. It has a very small reverse recovery charge compared with the Con-DTMOS and SG-DTMOS during switching operation owing to the heterojunction structure of the SHG-DTMOS, reducing the switching loss. The reverse recovery charge of the SHG-DTMOS was reduced by 65.83% and 73.45%, whereas the switching losses were reduced by 54.84% and 44.98%, respectively, compared with those of the Con-DTMOS and SG-DTMOS. In addition, the separate gate P^+^ shielding regions form a depletion region in the off state, preventing a high electric field from being applied to the P^+^ polysilicon, thereby reducing the reverse leakage current. As a result, the SHG-DTMOS can be used in high-frequency and high-voltage power systems for a short switching time and low switching loss when configuring a body diode.

## Figures and Tables

**Table 1:** Structure parameters of each device.

Parameter	Con-DTMOS	SG-DTMOS	SHG-DTMOS	Unit
Cell pitch	6	6	6	µm
Epi-layer thickness	13	13	13	µm
Gate trench width	2	2	2	µm
Source trench depth	1.5	1.5	1.5	µm
Channel length	0.7	0.7	0.7	µm
Channel gate length	1.45	0.9	0.9	µm
Gate oxide thickness	0.05	0.05	0.05	µm
L_ox_	0.05	0.05	0.05	µm
T_ox_	-	0.25	0.3	µm
W_PS_	-	-	0.6	µm
Doping concentration of N-drift (N type)	5 × 10^15^	5 × 10^15^	5 × 10^15^	cm^−3^
Doping concentration of CSL (N type)	2 × 10^16^	2 × 10^16^	2 × 10^16^	cm^−3^
Doping concentration of P^+^ region (P type)	5 × 10^18^	5 × 10^18^	5 × 10^18^	cm^−3^
Doping concentration of N^+^ polysilicon (N type)	1 × 10^21^	1 × 10^21^	1 × 10^21^	cm^−3^
Doping concentration of P^+^ polysilicon (P type)	-	-	5 × 10^18^	cm^−3^
Doping concentration of N^+^ substrate (N type)	5 × 10^19^	5 × 10^19^	5 × 10^19^	cm^−3^

**Table 2:** Static and dynamic characteristics of each devices.

Parameter	Con-DTMOS	SG-DTMOS	SHG-DTMOS	Unit
Breakdown voltage (BV)	1853	1852	1789	V
R_on-sp_ (@V_DS_ = 1 V)	4.74	5.55	5.45	mΩ∙cm^2^
E_mox_ (@V_DS_ = 1200 V)	2.09	2.09	1.33	MV∙cm^−1^
C_iss_ (@V_DS_ = 1200 V)	40.6	17.7	17.0	nF∙cm^−2^
C_rss_ (@V_DS_ = 1200 V)	11.06	2.05	2.5	pF∙cm^−2^
C_oss_ (@V_DS_ = 1200 V)	670	670	669	pF∙cm^−2^
Q_GD_	84.84	22.89	24.52	nC∙cm^−2^
Q_G_	826.1	372.8	362.5	nC∙cm^−2^
HF-FOM (R_on-sp_ × Q_GD_)	402.1	127.0	133.6	mΩ∙nC

**Table 3:** Body diode and switching characteristics of each devices.

Parameter	Con-DTMOS	SG-DTMOS	SHG-DTMOS	Unit
V_F_	2.88	2.88	1.88	V
t_rr_	63.7	52.7	46.9	ns
Q_rr_	2996	3804	1010	nC∙cm^−2^
T_off_	456	229	226	ns
T_on_	282	139	129	ns
T_SW_	738	368	355	ns
E_off_	0.737	0.572	0.552	mJ∙cm^−2^
E_on_	7.131	5.885	3.001	mJ∙cm^−2^
E_SW_	7.868	6.457	3.553	mJ∙cm^−2^

## Data Availability

Data are not available on a publicly accessible repository and they cannot be shared under request.
